# Construction and external validation of radiomics models to detect primary prostate cancer with machine learning: a multicenter study based on ^68^Ga-PSMA PET/CT

**DOI:** 10.1016/j.jncc.2025.12.002

**Published:** 2026-01-05

**Authors:** Jiaxian Chen, Xin Jiang, Guangjie Yang, Yongxiang Tang, Lin Qi, Minfeng Chen, Shuo Hu, Xiaomei Gao, Yu Gan, Mingxin Zhang, Shouzhen Chen, Yi Cai

**Affiliations:** a Department of Urology, Disorders of Prostate Cancer Multidisciplinary Team, National Clinical Research Center for Geriatric Disorders, Xiangya Hospital, Central South University, Changsha, China; b Department of Urology, The Affiliated Huaian No.1 People's Hospital of Nanjing Medical University, Huaian, China; c Department of Urology, Qilu Hospital, Cheeloo College of Medicine, Shandong University, Jinan, China; d Research Institute of Shandong University: Magnetic Field-free Medicine & Functional Imaging, Jinan, China; e Department of Nuclear Medicine, Affiliated Hospital of Qingdao University, Qingdao, China; f Department of PET Center, National Clinical Research Center for Geriatric Disorders, Xiangya Hospital, Central South University, Changsha, China; g Department of Pathology, Disorders of Prostate Cancer Multidisciplinary Team, National Clinical Research Center for Geriatric Disorders, Xiangya Hospital, Central South University, Changsha, China; h Department of Urology, Affiliated Hospital of Qingdao University, Qingdao, China

**Keywords:** ^68^Ga-PSMA PET/CT, Multicenter, Diagnosis, Radiomics, Machine learning

## Abstract

**Objective:**

Recent studies have shown that ^68^Ga-prostate-specific membrane antigen (^68^Ga-PSMA) positron emission tomography/computed tomography (PET/CT) can open a non-invasive diagnostic pathway in prostate cancer (PCa). However, the relatively small number of enrolled patients in these studies limits statistical validation and causes bias to some extent. In addition, the performance of ^68^Ga-PSMA PET/CT radiomics analysis in detecting PCa has not been widely evaluated. Hence, the present multicenter study endeavored to develop and validate radiomics models based on ^68^Ga-PSMA PET/CT for the detection of primary PCa in a relatively larger cohort.

**Methods:**

This study enrolled consecutive patients with suspected PCa who underwent systematic biopsy (SB) and PSMA PET/CT-targeted biopsy (TB) after ^68^Ga-PSMA PET/CT in three medical centers. The whole prostate gland was adopted as the volume of interest (VOI). Eight machine learning (ML) algorithms were utilized to develop models with selected radiomics features separately, after which the best-performing radiomics model was integrated with maximum standardized uptake value (SUVmax) to create the combined model. The receiver operating characteristic (ROC) curves and area under the curve (AUC) value, accuracy, sensitivity, specificity, positive predictive value (PPV), and negative predictive value (NPV) were calculated for each model.

**Results:**

Overall, 609 patients were recruited, including 195 patients with benign prostate diseases (BPD), 30 patients with clinically insignificant prostate cancer (ciPCa, Gleason score [GS] = 3 + 3), and 384 patients with clinically significant prostate cancer (csPCa, GS ≥ 3 + 4). For csPCa prediction, the radiomics model developed by the eXtreme Gradient Boosting (XGBoost) algorithm demonstrated the best performance. After integrating with SUVmax, the combined model achieved the highest AUC of 0.921 in internal validation cohort. External validation cohort 1 and 2 also showed promising results with AUC of 0.906 and 0.898, respectively. For PCa prediction, the XGBoost algorithm combined with SUVmax also peformed well in three validation cohorts with the AUC ranging from 0.860 to 0.918.

**Conclusions:**

This is the largest multicenter study to date, providing a noninvasive and quantitative method based on ^68^Ga-PSMA PET/CT radiomics analysis modeled with ML for predicting PCa. This method has the potential to gauge the risk of PCa before biopsy.

## 1 Introduction

Men suspected of harboring prostate cancer (PCa) due to an elevated prostate-specific antigen (PSA) level or an abnormal digital rectal examination (DRE) are often offered a standard transrectal ultrasonography (TRUS)-guided biopsy of the prostate. However, this approach is associated with the over-detection of benign prostatic hyperplasia (BPD) and the under-detection of malignant tumors. Moreover, it is also related to troublesome complications, such as bleeding, pain, and infection, and poses a high economic and psychological burden to patients.^1^ A non-invasive imaging approach to distinguish PCa from BPD is urgently needed in clinical practice to prevent patients from unnecessary biopsies.

Prostate multi-parametric magnetic resonance imaging (mpMRI) has been the most commonly used imaging technique in PCa detection and risk stratification for years since the development of the Prostate Imaging Reporting and Data System (PI-RADS) scoring system.^2^ Patients with prostate lesions classified as PI-RADS scores 3–5 are recommended to undergo biopsies by a urologist.^3^^,^^4^ However, the detection rates of csPCa were 12 %, 60 %, and 83 % in patients with PI-RADS scores of 3–5, respectively.^5^ Besides, it has been reported that approximately 10 % of PCa cases would be missed by mpMRI.^6^ Generally, the noninvasive diagnosis of PCa is still limited by the diagnostic accuracy of mpMRI, especially the low specificity.

In the last two decades, molecular imaging with ^68^Ga-prostate-specific membrane antigen (^68^Ga-PSMA) positron emission tomography/computed tomography (PET/CT) has shown superiority in staging high-risk PCa after biopsy or biochemical recurrence after local treatment.^7^ An improvement in the sensitivity and specificity of ^68^Ga-PSMA PET/CT compared with mpMRI raises the question of whether it can complement or even replace mpMRI to avoid unnecessary prostate biopsy. To date, numerous studies have confirmed that the standardized uptake value (SUV) and other semi-quantitative parameters of suspected prostate lesions derived from ^68^Ga-PSMA PET/CT help differentiate PCa from BPD.^8^, ^9^, ^10^, ^11^, ^12^, ^13^, ^14^, ^15^, ^16^, ^17^ A recent study reported that the SUVmax ≥ 9.0 had a 100 % specificity in detecting clinically significant prostate cancer (csPCa) for patients with a PI-RADS score of 4 or 5.^9^^,^^18^ Meissner et al. firstly reported 25 patients with highly suspected csPCa through mpMRI and ^68^Ga-PSMA PET who received radical prostatectomy (RP) without prior biopsy diagnosis.^19^ The use of PSMA PET/CT has heralded a new era in noninvasive diagnosis of PCa.

However, the patient cohorts enrolled in these studies were relatively small from single medical center, which may limit statistical validation and cause bias to some extent.^8^, ^9^, ^10^, ^11^, ^12^, ^13^, ^14^, ^15^, ^16^, ^17^ Therefore, multicenter studies with larger sample sizes are urgently needed. Besides, semi-quantitative parameters, such as SUVmax, may be influenced by injection dose, the time interval between injection and scan, and other factors, limiting their generalizability, especially in different medical centers.^20^ Thus, radiomics analysis combined with the machine learning (ML) method may provide more robust and credible results in the era of PCa detection, as radiomics can extract high-throughput data from original images. The diagnostic value of ^68^Ga-PSMA PET/CT radiomics analysis in patients with a clinical suspicion of PCa has not been fully explored.

Hence, we perform this multicenter study, with the help of the Chestnut electronic data capture system (EDC),^18^ sought to develop radiomics models based on ^68^Ga-PSMA PET/CT combined with different ML algorithms and evaluate their diagnostic performance for the detection of PCa in a relatively larger cohort.

## 2 Materials and methods

### 2.1 Study population and endpoint

We retrospectively reviewed all patients at Xiangya Hospital of Central South University (Center 1) who underwent ^68^Ga-PSMA PET/CT examination between July 2019 and July 2023 due to suspicion of PCa and another two cohorts from Qilu Hospital of Shandong University (Center 2) and Qingdao University Medical College (Center 3). Biopsy-proven BPD patients or PCa patients who underwent systematic biopsy (SB) and necessary PET/CT-targeted biopsy (TB) for PET/CT-positive patients were eligible for the study. The biopsy criteria of this study can be found in the Supplementary materials. The exclusion criteria were as follows: (a) patients whose biopsy was taken before PET/CT or the interval time between PET/CT examination and biopsy was longer than one month; (b) patients whose PET/CT was positive but underwent systematic biopsy only without PET/CT fusion-targeted biopsy; (c) patients who received radiotherapy, chemotherapy, androgen deprivation therapy, or prostate surgery before PET/CT. The flowchart of the patient selection process was shown in Supplementary Fig. 1. Data were also collected from all enrolled patients who had underwent the mpMRI examination at Center 1 and 2. Detailed information of mpMRI examination and image evaluation was shown in the Supplementary materials.

The primary endpoint was to establish and validate a radiomics model to discriminate csPCa and non-csPCa patients (namely BPD and ciPCa). Additionally, the performance of the PI-RADS score for csPCa prediction was also included as subgroup analysis. The secondary endpoint of this study was the construction and validation of a ^68^Ga-PSMA PET/CT-based radiomics model to distinguish PCa patients from BPD patients. For each endpoint, eligible patients from Center 1 were randomly divided into a training cohort and an internal validation cohort at a ratio of 1:1. Patients from Center 2 and Center 3 were recruited as an external validation cohort 1 and 2, respectively.

### 2.2 ^68^Ga-PSMA PET/CT imaging and volume of interest segmentation

All enrolled patients in three medical centers underwent ^68^Ga-PSMA PET/CT examinations before prostate biopsy. Each patient received a ^68^Ga-PSMA of 1.8–2.2 MBq/kg as the European Association of Nuclear Medicine Nuclear (EANM) / Medicine and Molecular Imaging / Society of Nuclear Medicine and Molecular Imaging (SNMMI) guidelines suggested and subsequent scans after 40 ± 10 min using a PET/CT scanner. Original images from three centers were collected and two expericenced nuclear medicine physicians independently reevaluated all images blinded to the patient’s clinical data. The positive lesions were defined as follows: (a) focal transition zone activity visually twice above background; (b) focal peripheral zone activity (no minimum intensity); (c) intense uptake (visual very high intensity or SUVmax > 12).^21^ Negative PET/CT scans were defined as follows: (a) no dominant intraprostatic activity higher than that of surrounding prostate tissue; (b) diffuse transition zone activity or symmetrical central zone activity that does not extend to the prostate margin on CT. The SUVmax of the dominant lesion (defined as the highest focal uptake on PET images) in the prostate gland for PET/CT-positive patients and the prostate gland background for PET/CT-negative patients were recorded for further analysis.

The whole prostate gland was adopted as the volume of interest (VOI). PET images were loaded into ITK-SNAP software (version 3.8.0) and viewed side-by-side with axial CT images of each patient for segmentation, allowing VOIs to be drawn directly on both PET and co-registered CT images. A radiologist who was blinded to the pathological results manually completed the tissue segmentation, and all VOIs were reviewed by a second observer. Consensus was reached by joint revision if necessary.

### 2.3 Biopsy protocol and histopathological examination

Experienced urological surgeons conducted a standard TRUS-targeted transperineal 12-core biopsy for every enrolled subject, and if necessary, additional PSMA PET/CT-guided TB for PET/CT-positive patients (details shown in the Supplementary materials).^22^^,^^23^ All biopsy specimens were reviewed by experienced uropathologists based on the Gleason score (GS) and International Society of Urological Pathology (ISUP) grade group (GG). Patients with a GS ≥ 3 + 3 were diagnosed with PCa, while others such as benign prostatic hyperplasia and inflammation were classified as BPD. csPCa was defined as GS ≥ 3 + 4, while GS = 3 + 3 was considered insignificant prostate cancer (ciPCa).^24^

### 2.4 Radiomics feature extraction and selection

Radiomics features were extracted from the VOIs of both PSMA PET and CT images following the guidelines of the Image Biomarker Standardization Initiative.^25^ Overall, 2353 radiomic features (1223 features from CT images and 1130 from PET images) were extracted from the original VOIs without resampling, using the feature package PyRadiomics (github.com/Radiomics/pyradiomics) in Python. Details of the radiomics features extraction was illustrated in the Supplementary materials. After eliminating redundant and irrelevant features using the classic minimum redundancy maximum relevance (mRMR) algorithm, 30 features were reserved for further analysis.^26^ Next, the least absolute shrinkage and selection operator (LASSO) algorithm was employed to select significant distinguishable features.

### 2.5 Model construction and validation

**Fig. 1:**
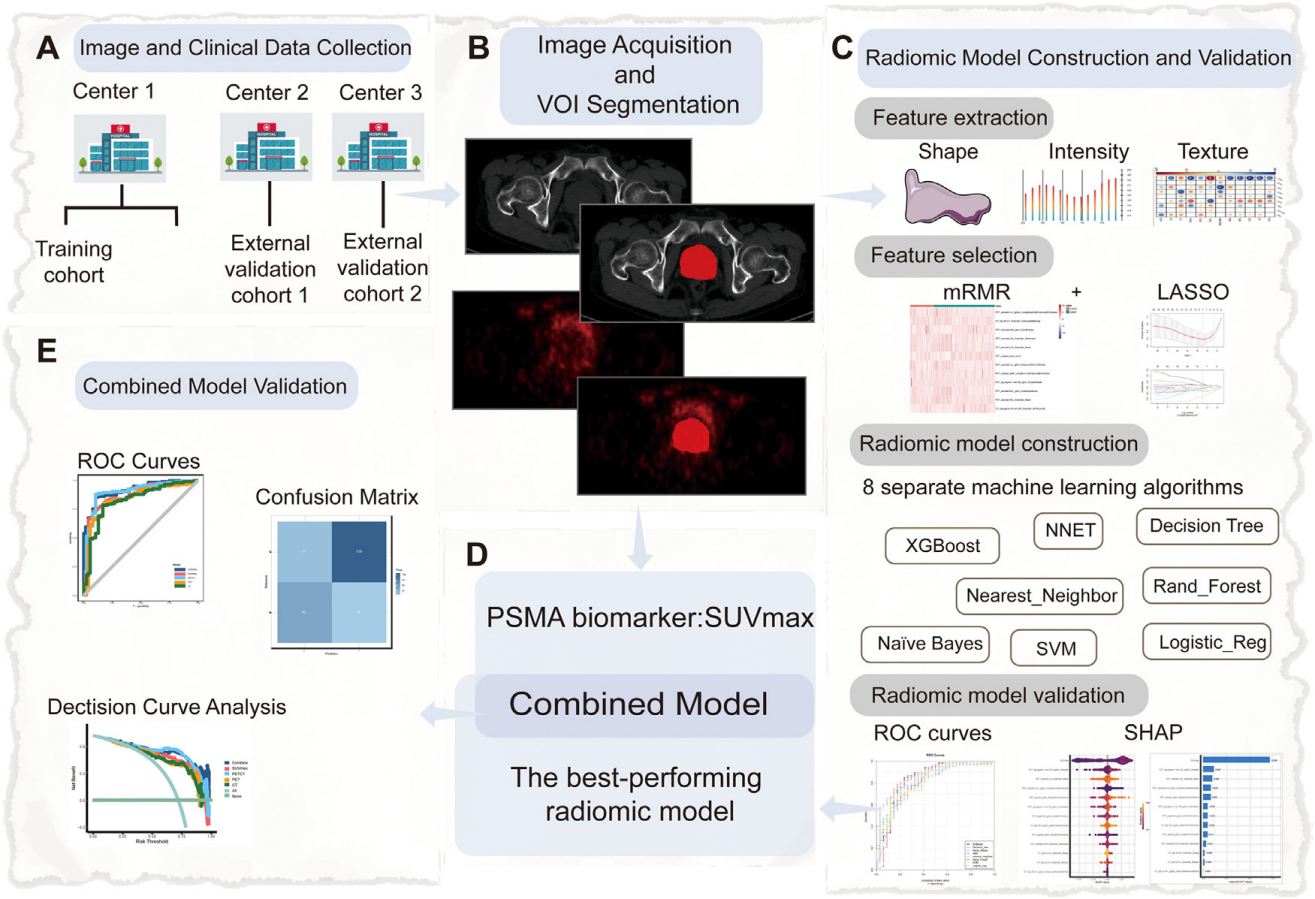
Workflow of the study. (A) Collection of clinical data and images. (B) ^68^Ga-PSMA PET/CT imaging and VOI segmentation. (C) Radiomics models construction and validation: Radiomics features were extracted from the VOIs of both PSMA PET and CT images. The mRMR and LASSO algorithm were used to select radiomics features. Then 8 separate algorithms were used to develop radiomics models. (D) Construction of the combined model by integrating the best-performing radiomics with SUVmax. (E) Validation of the combined model across different cohorts. LASSO, least absolute shrinkage and selection operator; mRMR, minimum redundancy maximum relevance; PET/CT, positron emission tomography/computed tomography; PSMA, prostate-specific membrane antigen; ROC, receiver operating characteristic; SHAP, SHapley Additive exPlanations; SUVmax, maximum standardized uptake value; VOI, volume of interest.

After radiomics features were selected by the mRMR and LASSO algorithms, eight ML algorithms were utilized to develop radiomics models with 10-fold cross-validation approach, respectively, namely eXtreme Gradient Boosting (XGBoost), decision tree, Naïve Bayes, Neural Network (NNET), K-nearest neighbor (KNN), random forest (RF), logistic regression (LR), and support vector machine (SVM). The receiver operating characteristic (ROC) curves and AUC values were used to assess the performance for each radiomics model. We used SHapley Additive exPlanations (SHAP) to visualize and analyze the radiomics model with the highest AUC value. Then the best-performing radiomics model was integrated with SUVmax to create the combined model. Confusion Matrix analysis was used to estimate the combined model performance for each study endpoint, including AUC, accuracy, sensitivity, specificity, positive predictive value (PPV) and negative predictive value (NPV). The net benefit of the combined model was determined using decision curve analysis (DCA). Workflow of the study was illustrated in Fig. 1.

### 2.6 Statistical analysis

Statistical analyses were conducted with Python 3.7.3 (https://www.python.org/) and R software 4.2.1 (https://www.r-project.org/). Continuous variables with skewed distribution were expressed as the median (interquartile range [IQR]), while the mean ± standard deviation (SD) was used for normal distribution. Categorical variables were presented as frequencies (percentages).

## 3 Results

### 3.1 Clinical characteristics

A total of 609 patients who met the inclusion and exclusion criteria were enrolled. The baseline clinical characteristics of the patients from three centers were summarized in Table 1. The detailed information of different cohorts for csPCa and PCa prediction was shown in Supplementary Table 1 and Supplementary Table 2, respectively.

**Table 1:** Baseline clinical characteristics of the enrolled patients.

Characteristic	Center 1	Center 2	Center 3
Number of patients	349	192	68
Age at biopsy, mean ± SD, years	66.6 ± 7.9	67.9 ± 8.4	69.1 ± 8.1
PSA at biopsy, median (IQR), ng/mL	16.1 (8.3–39.7)	7.2 (4.5–17.8)	27.4 (12.5–95.6)
PSA≤10ng/ml, No. (%)	106 (30.4)	121 (63.0)	15 (22.0)
10ng/ml<PSA≤20ng/ml, No. (%)	95 (27.2)	28 (14.6)	18 (26.5)
PSA>20ng/ml, No. (%)	148 (42.4)	43 (22.4)	35 (51.5)
DRE, No. (%)			
Abnormal	192 (55.0)	90 (46.9)	44 (64.7)
Normal	157 (45.0)	102 (53.1)	24 (35.3)
SUVmax of dominant lesion of suspicion, median (IQR)	9.7 (5.4–19.5)	7.9 (4.4–19.5)	20.5 (12.3–41.3)
Pathologic results, No. (%)			
BPD	97 (27.8)	84 (43.7)	14 (20.6)
PCa	252 (72.2)	108 (56.3)	54 (79.4)
ISUP grade group, No. (%)			
GG 1	20 (7.9)	6 (5.6)	4 (7.4)
GG 2	46 (18.3)	16 (14.8)	7 (13.0)
GG 3	33 (13.1)	19 (17.6)	4 (7.4)
GG 4	52 (20.6)	32 (29.6)	19 (35.2)
GG 5	101 (40.1)	35 (32.4)	20 (37.0)

Abbreviations: BPD, benign prostate diseases; DRE, digital rectal examination; GG, grade group; IQR, interquartile range; ISUP, International Society of Urological Pathology; PCa, prostate cancer; PSA, prostate-specific antigen; SD, standard deviation; SUVmax, maximum standardized uptake value.

### 3.2 csPCa prediction

#### 3.2.1 Radiomics model

For csPCa prediction, the 12 most relevant radiomics features were selected and used for model construction with eight ML algorithms (details shown in the Supplementary materials). The area under curve (AUC) value of eight ML algorithms in predicting csPCa was summarized in Table 2. The XGBoost algorithm achieved the highest AUC of 0.902 in the training cohort. It also demonstrated strong predictive performance in the internal validation cohort and two external validation cohorts, with AUC values of 0.901, 0.832, and 0.849, respectively. In the radiomics model trained by the XGBoost algorithm, individual SHAP value and the effects on the prediction probability of each selected radiomics feature were illustrated in the Supplementary Fig. 2. Among the 12 selected features, “PET_wavelet. LHL_firstorder_Mean” had the strongest correlation with the presence of csPCa.

**Table 2:** The AUC of radiomics models developed by eight machine learning algorithms for csPCa prediction.

Machine learning algorithm	AUC (95 % CI)			
	Training cohort (*n* = 175)	Internal validation cohort (*n* = 174)	External validation cohort 1 (*n* = 192)	External validation cohort 2 (*n* = 68)
XGBoost	0.902 (0.854–0.950)	0.901 (0.854–0.947)	0.832 (0.779–0.890)	0.849 (0.744–0.954)
Decision Tree	0.828 (0.759–0.897)	0.803 (0.734–0.872)	0.769 (0.702–0.836)	0.777 (0.651–0.903)
Logistic_Reg	0.834 (0.767–0.901)	0.819 (0.755–0.883)	0.764 (0.698–0.83)	0.763 (0.653–0.873)
NNET	0.833 (0.768–0.898)	0.827 (0.764–0.889)	0.733 (0.663–0.804)	0.742 (0.611–0.874)
Naïve Bayes	0.850 (0.791–0.908)	0.836 (0.774–0.898)	0.770 (0.704–0.836)	0.782 (0.651–0.913)
Nearest_Neighbor	0.817 (0.753–0.881)	0.804 (0.737–0.872)	0.738 (0.668–0.808)	0.754 (0.633–0.876)
Rand_Forest	0.868 (0.808–0.927)	0.868 (0.812–0.923)	0.814 (0.754–0.874)	0.819 (0.703–0.935)
SVM	0.825 (0.756–0.894)	0.816 (0.750–0.883)	0.753 (0.684–0.823)	0.764 (0.615–0.914)

Abbreviations: AUC, area under the receiver operating characteristic curve; CI, confidence interval; csPCa, clinically significant prostate cancer; Logistic_Reg, logistic regression; Nearest_Neighbor, K-nearest neighbor; NNET, neural network; Rand_Forest, random forest; SVM, support vector machine; XGBoost, eXtreme Gradient Boosting.

#### 3.2.2 Combined model

**Fig. 2:**
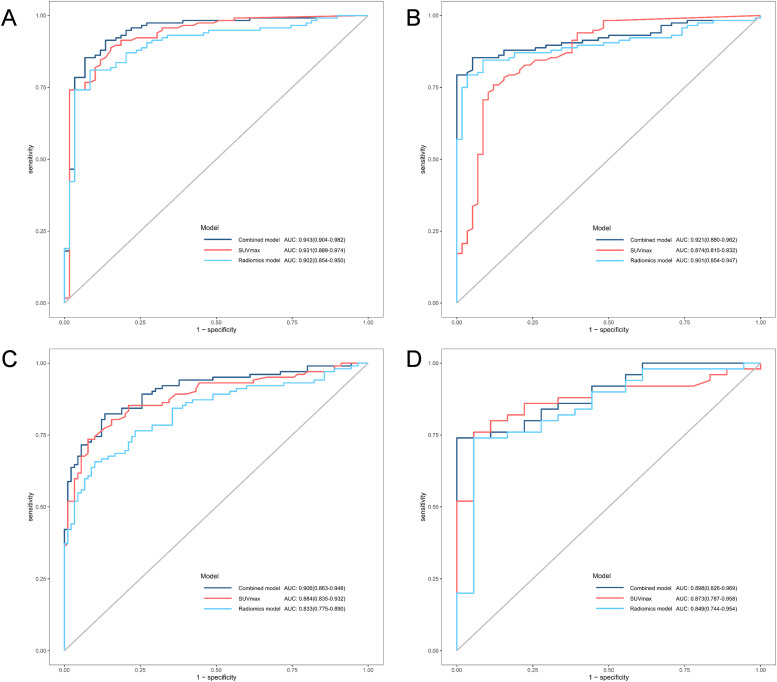
Receiver operating characteristic curves of the radiomics model, SUVmax and combined model across different cohorts for clinically significant prostate cancer prediction. (A) Training cohort. (B) Internal validation cohort. (C) External validation cohort 1. (D) External validation cohort 2. The AUC was presented as mean (95 % CI). AUC, area under curve; SUVmax, maximum standardized uptake value.

**Fig. 3:**
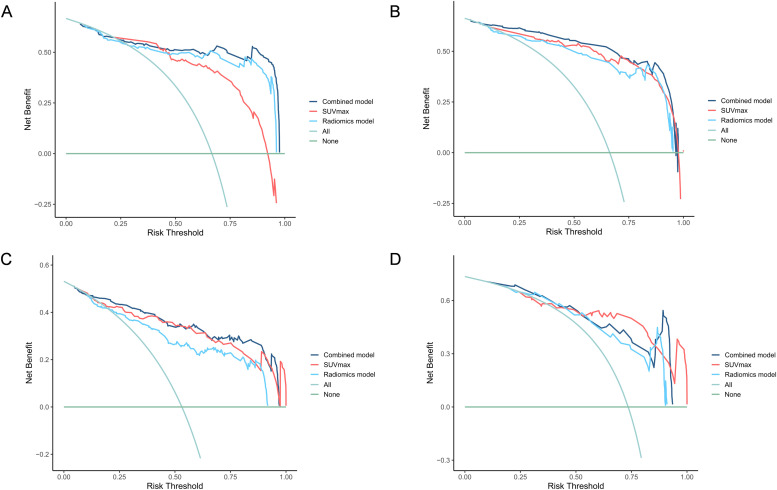
Decision curve analysis curves of the radiomics model, SUVmax and combined model across different cohorts for clinically significant prostate cancer prediction. (A) Training cohort. (B) Internal validation cohort. (C) External validation cohort 1. (D) External validation cohort 2. SUVmax, maximum standardized uptake value.

Subsequently, a joint model was developed by integrating the radiomics model trained by the XGBoost algorithm and the SUVmax. The predictive performance of the radiomics model, SUVmax and combined model across different cohorts was summarized in Table 3. The combined model demonstrated superior performance across all cohorts compared to the individual model alone. The ROC curves of different models discriminating csPCa and non-csPCa patients were shown in Fig. 2. Specifically, in the internal validation cohort, the combined model achieved an AUC of 0.921, with a sensitivity, specificity, PPV, and NPV of 85.3 %, 94.8 %, 97.1 %, and 76.4 %, respectively. In external validation cohort 1, the integrated model yielded an AUC of 0.906, accompanied by sensitivity of 82.4 %, specificity of 86.7 %, PPV of 87.5 %, and NPV of 81.2 %. In external validation cohort 2, the integrated model yielded an AUC of 0.898, with a sensitivity, specificity, PPV, and NPV of 74.0 %, 100.0 %, 100.0 %, and 58.1 %. Confusion Matrix analysis of the combined model across different cohorts for PCa prediction was shown in Supplementary Fig. 3. As shown in Fig. 3, DCA was performed to compare the clinical utility of different prediction models in assisting prostate biopsy decisions. As expected, the combined model achieved obviously higher net benefits than biopsy-all strategy for risk thresholds over 25 %.

**Table 3:** The performance of the radiomics model, SUVmax and combined model across different cohorts for csPCa prediction.

Cohort	Model	AUC (95 % CI)	Accuracy, %	Sensitivity, %	Specificity, %	PPV, %	NPV, %
Training cohort (*n* = 175)	Radiomics model	0.902 (0.854–0.950)	84.6	81.0	91.5	94.9	71.1
	SUVmax	0.931 (0.889–0.974)	87.4	88.8	84.7	92.0	79.4
	Combined model	0.943 (0.904–0.982)	88.0	85.3	93.2	96.1	76.4
Internal validation cohort (*n* = 174)	Radiomics model	0.901 (0.854–0.947)	86.8	84.5	91.4	95.1	74.6
	SUVmax	0.874 (0.815–0.932)	79.9	75.9	87.9	92.6	64.6
	Combined model	0.921 (0.880–0.962)	88.5	85.3	94.8	97.1	76.4
External validation cohort 1 (*n* = 192)	Radiomics model	0.833 (0.775–0.890)	77.1	65.7	90.0	88.2	69.8
	SUVmax	0.884 (0.835–0.932)	82.3	73.5	92.2	91.5	75.5
	Combined model	0.906 (0.863–0.948)	84.4	82.4	86.7	87.5	81.2
External validation cohort 2 (*n* = 68)	Radiomics model	0.849 (0.744–0.954)	79.4	74.0	94.4	97.4	56.7
	SUVmax	0.873 (0.787–0.958)	80.9	76.0	94.4	97.4	58.6
	Combined model	0.898 (0.826–0.969)	80.9	74.0	100.0	100.0	58.1

Abbreviations: AUC,area under the receiver operating characteristic curve; CI, confidence interval; csPCa, clinically significant prostate cancer; NPV, negative predictive value; PPV, positive predictive value; SUVmax, maximum standardized uptake value.

#### 3.2.3 Subgroup analysis of PI-RADS score for csPCa prediction

The baseline clinical characteristics of patients who completed mpMRI examination was shown in Supplementary Table 3. Among the 349 patients recruited at Center 1, 189 completed the mpMRI examination. With a PI-RADS score of ≥3 used as the cutoff value, the AUC for mpMRI in detecting csPCa was 0.535 as shown in Table 4. When using a PI-RADS score of ≥4 as the cutoff value, the AUC of mpMRI for diagnosing csPCa was 0.823, with a sensitivity, specificity, PPV, NPV of 83.6 %, 81.0 %, 85.9 % and 78.0 %, respectively. A total of 192 patients at Center 2 completed both PSMA PET/CT and mpMRI examination. The AUC for mpMRI in detecting csPCa was 0.685 when a PI-RADS score ≥3 was applied as the cutoff value, with a sensitivity, specificity, PPV, NPV of 89.2 %, 46.6 %, 65.9 % and 77.7 %. While with a PI-RADS score of ≥4 used as the cutoff value, the AUC in distinguishing csPCa reached 0.720, accompanied by sensitivity of 69.6 %, specificity of 74.4 %, PPV of 75.5 %, and NPV of 68.6 %.

**Table 4:** The performance of the PI-RADS score for csPCa prediction with different cutoff value.

	Cutoff value	AUC	Accuracy, %	Sensitivity, %	Specificity, %	PPV, %	NPV, %
Center 1	PI-RADS ≥ 3	0.535	60.8	98.1	8.8	60.0	77.7
	PI-RADS ≥ 4	0.823	81.3	83.6	81.0	85.9	78.0
Center 2	PI-RADS ≥ 3	0.685	69.3	89.2	46.6	65.9	77.7
	PI-RADS ≥ 4	0.720	71.9	69.6	74.4	75.5	68.3

Abbreviations: AUC, area under the receiver operating characteristic curve; mpMRI, multi-parameter magnetic resonance imaging; NPV, negative predictive value; PI-RADS, Prostate Imaging Reporting and Data System; PPV, positive predictive value.

### 3.3 PCa prediction

#### 3.3.1 Radiomics model

For csPCa prediction, the 12 most relevant radiomics features were selected and used for model construction with eight ML algorithms (details shown in the Supplementary materials). The AUC of each ML algorithm model for PCa prediction are summarized in Table 5. The XGBoost algorithm achieved the highest AUC of 0.911 in the training cohort. The prediction models in three validation cohorts showed similar trends, with the XGBoost algorithm also achieving the highest AUC. In the radiomics model trained by the XGBoost algorithm, individual SHAP value and the effects on the prediction probability of each selected radiomics feature were illustrated in the Supplementary Fig. 4. Among the 12 selected features, “PET_wavelet. LHL_firstorder_Mean” still had the strongest correlation with the presence of PCa.

**Table 5:** The AUC of radiomics models developed by eight machine learning algorithms for PCa prediction.

Machine learning algorithm	AUC (95 % CI)			
	Training cohort (*n* = 175)	Internal validation cohort (*n* = 174)	External validation cohort 1 (*n* = 192)	External validation cohort 2 (*n* = 68)
XGBoost	0.911 (0.807–0.955)	0.901 (0.846–0.956)	0.846 (0.791–0.902)	0.849 (0.741–0.957)
Decision Tree	0.837 (0.771–0.903)	0.838 (0.773–0.904)	0.779 (0.712–0.847)	0.770 (0.649–0.891)
Logistic_Reg	0.824 (0.755–0.894)	0.830 (0.763–0.896)	0.785 (0.719–0.850)	0.772 (0.646–0.899)
NNET	0.841 (0.779–0.902)	0.832 (0.764–0.900)	0.756 (0.688–0.825)	0.776 (0.652–0.901)
Naïve Bayes	0.864 (0.806–0.922)	0.835 (0.769–0.901)	0.786 (0.722–0.850)	0.782 (0.647–0.918)
Nearest_Neighbor	0.809 (0.737–0.880)	0.807 (0.730–0.883)	0.767 (0.701–0.833)	0.774 (0.664–0.884)
Rand_Forest	0.884 (0.835–0.932)	0.877 (0.823–0.931)	0.812 (0.751–0.874)	0.804 (0.691–0.917)
SVM	0.848 (0.779–0.917)	0.806 (0.730–0.882)	0.785 (0.718–0.852)	0.782 (0.665–0.899)

Abbreviations: AUC, area under the receiver operating characteristic curve; CI, confidence interval; Logistic_Reg, logistic regression; Nearest_Neighbor, K-nearest neighbor; NNET, neural network; PCa, prostate cancer; Rand_Forest, random forest; SVM, support vector machine; XGBoost, eXtreme Gradient Boosting.

#### 3.3.2 Combined model

**Fig. 4:**
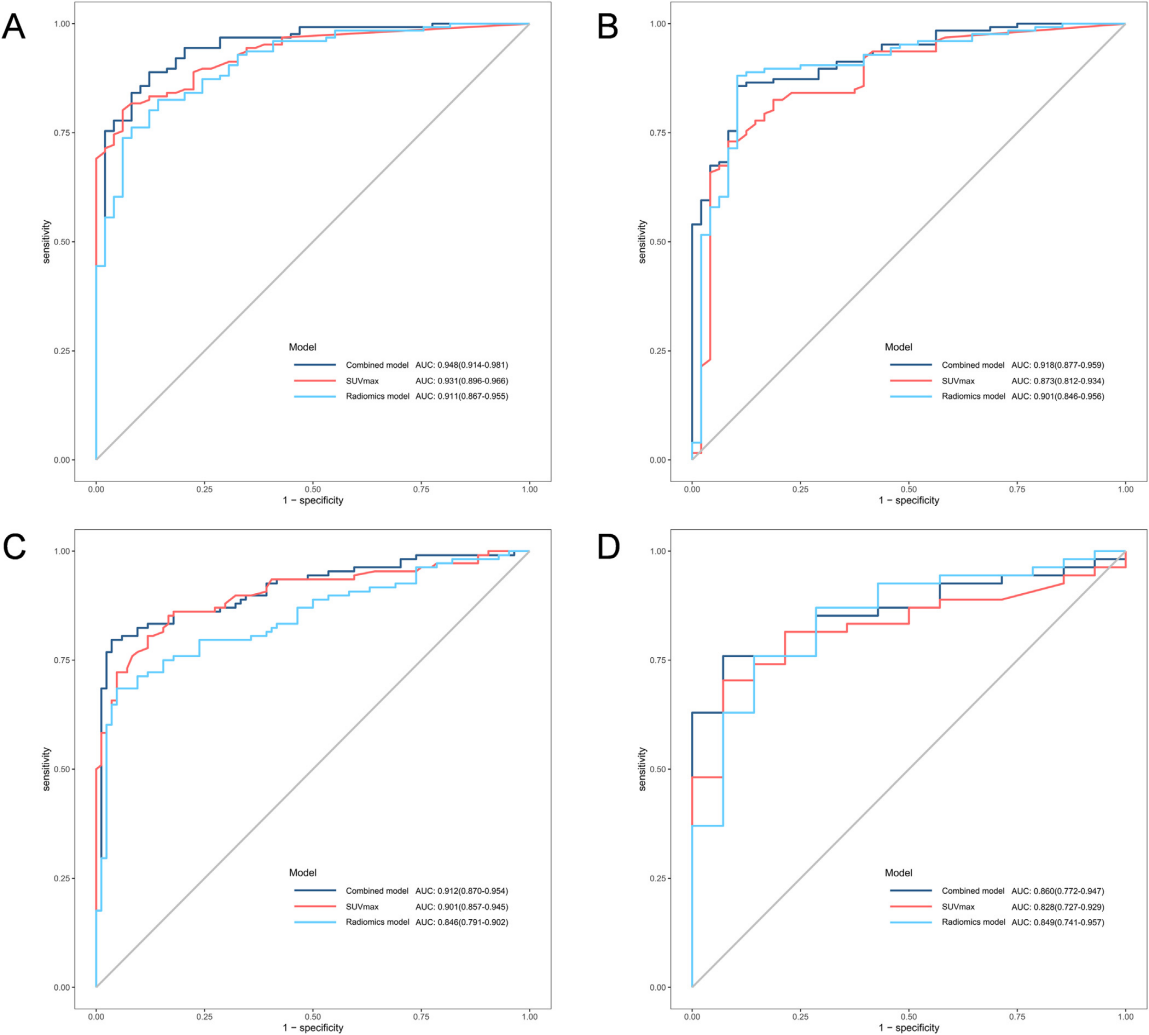
Receiver operating characteristic curves of the radiomics model, SUVmax and combined model across different cohorts for prostate cancer prediction. (A) Training cohort. (B) Internal validation cohort. (C) External validation cohort 1. (D) External validation cohort 2. The AUC was presented as mean (95 % CI). AUC, area under curve; SUVmax, maximum standardized uptake value.

**Fig. 5:**
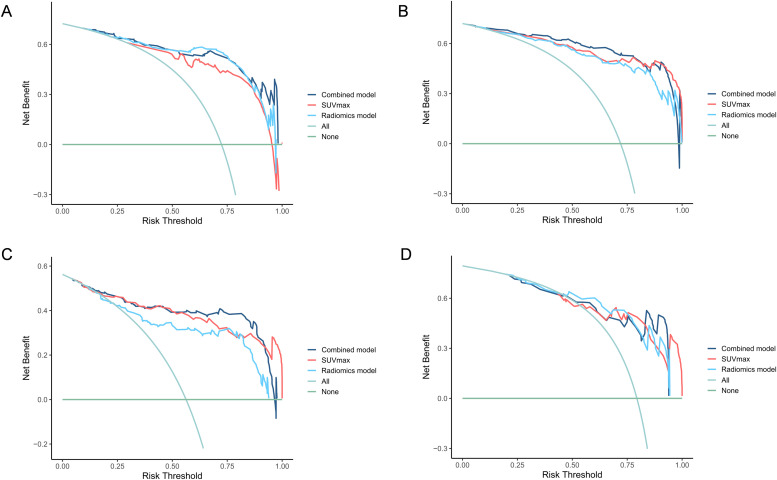
Decision curve analysis curves of the radiomics model, SUVmax and combined model across different cohorts for prostate cancer prediction. (A) Training cohort. (B) Internal validation cohort. (C) External validation cohort 1. (D) External validation cohort 2. SUVmax, maximum standardized uptake value.

Following the same strategy as described above, the SUVmax was combined with radiomics model to form the final model. The predictive performance of the each model across different cohorts was summarized in Table 6. And the ROC curves were shown in Fig. 4. In the internal validation cohort, the combined model achieved an AUC of 0.918, with a sensitivity, specificity, PPV, and NPV of 85.7 %, 89.6 %, 95.6 %, and 70.5 %, respectively. In external validation cohort 1, the integrated model yielded an AUC of 0.912, accompanied by sensitivity of 79.6 %, specificity of 96.4 %, PPV of 96.6 %, and NPV of 78.6 %. In external validation cohort 2, the combined model yielded an AUC of 0.860, with a sensitivity, specificity, PPV, and NPV of 75.9 %, 92.9 %, 97.6 %, and 50.0 %. Confusion Matrix analysis of the combined model across different cohorts for PCa prediction was shown in Supplementary Fig. 5. As shown in Fig. 5, the combined model achieved obviously higher net benefits than biopsy-all strategy for risk thresholds over 40 %.

**Table 6:** The performance of the radiomics model, SUVmax and combined model across different cohorts for PCa prediction.

Cohort	Model	AUC (95 %CI)	Accuracy, %	Sensitivity, %	Specificity, %	PPV, %	NPV, %
Training cohort (*n* = 175)	Radiomics model	0.911 (0.867–0.955)	83.4	82.5	85.7	93.7	65.6
	SUVmax	0.931 (0.896–0.966)	84.0	80.2	93.9	97.1	64.8
	Combined model	0.948 (0.914–0.981)	88.6	88.9	87.8	94.9	75.4
Internal validation cohort (*n* = 174)	Radiomics model	0.901 (0.846–0.956)	88.5	88.1	89.6	95.7	74.1
	SUVmax	0.873 (0.812–0.934)	78.2	73.0	91.7	95.8	56.4
	Combined model	0.918 (0.877–0.959)	86.8	85.7	89.6	95.6	70.5
External validation cohort 1 (*n* = 192)	Radiomics model	0.846 (0.791–0.902)	80.2	68.5	95.2	94.9	70.2
	SUVmax	0.901 (0.857–0.945)	83.9	80.6	88.1	89.7	77.9
	Combined model	0.912 (0.870–0.954)	87.0	79.6	96.4	96.6	78.6
External validation cohort 2 (*n* = 68)	Radiomics model	0.849 (0.741–0.957)	77.9	75.9	85.7	95.3	48.0
	SUVmax	0.828 (0.727–0.929)	75.0	70.4	92.9	97.4	44.8
	Combined model	0.860 (0.772–0.947)	79.4	75.9	92.9	97.6	50.0

Abbreviations: AUC, area under the receiver operating characteristic curve; CI, confidence interval; NPV, negative predictive value; PCa, prostate cancer; PPV, positive predictive value; SUVmax, maximum standardized uptake value.

## 4 Discussion

To the best of our knowledge, this is the first multicenter study to explore the noninvasive diagnostic value of ^68^Ga-PSMA PET/CT radiomics for PCa in such a large-scale cohort including 195 patients with BPD and 414 patients with PCa, totally.

The radiomics model trained by the XGBoost algorithm exhibited the highest diagnostic accuracy. To improve the interpretability of radiomics model, the SHAP analysis was employed and “PET_wavelet. LHL_firstorder_Mean” was identified as the top-ranked radiomic feature for the prediction of both csPCa and PCa. It simultaneously captures information about both metabolic activity and lesion texture. This feature, which is the mean intensity of pixel values in the lesion area on a PSMA PET image after a wavelet transform at a specific scale, serves as a robust indicator for predicting prostate cancer.

When radiomics features was integrated with SUVmax, the combined model achieved higher AUC ranging from 0.898 to 0.921 across three validation cohorts for csPCa prediction. For PCa prediction, the combined model demonstrated similar trends. In this multicenter study, baseline characteristics demonstrated obvious statistical difference in key features between validation cohorts, which suggested heterogeneity among the patients recruited from three medical centers. But the combined models maintained high accuracy across different validation cohort. This undoubtedly served as strong evidence of the model’s superior generalization capability and broader applicability. Additionally, DCA curves revealed a superior net benefit for the combined model over a broad threshold range, highlighting its clinical utility. mpMRI has shown higher sensitivity for csPCa over traditional systematic biopsy in the PROMIS and PRECISION trials.^27^^,^^28^ However, mpMRI is still far from the optimal triage test due to high false-positive rate.^29^ The application of PI-RADS relies on imaging quality and requires the high-level expertise of readers.^30^^,^^31^ Therefore, PCa detection by mpMRI is highly dependent on the imaging quality and the radiologist’s experience. This is consistent with the findings of the mpMRI subgroup analysis in our study, which demonstrated that mpMRI was inferior to that of the model based on PSMA PET/CT for csPCa prediction.

The utilization of PSMA PET/CT has increasingly expanded from post-diagnostic to pre-diagnostic phases in prostate cancer management in recent years. It was reported that PSMA PET/CT with a SUVmax cutoff of 8 demonstrated a good accuracy equal to 100 % of ISUP GG≥3 PCa.^32^ Also, PSMA PET/CT-targeted prostate biopsies performed better than mpMRI-targeted biopsies.^33^ Compared to mpMRI, PSMA PET/CT offers higher specificity in detecting csPCa lesions, which enables it to play a critical role in the follow-up of patients under active surveillance.^34^ In conclusion, PSMA PET/CT has emerged as a useful tool complementing and even replacing the role of mpMRI in the diagnosis and initial staging of PCa.^7^ In comparison to sequential assessment with prostate mpMRI followed by PSMA PET/CT, utilizing PSMA PET/CT as a single procedure for men at high risk for csPCa offers advantages in consideration of the cost-effectiveness. Because PSMA PET/CT is not only more accurate in detecting primary lesions but also serves as a whole-body examination to assess systemic tumor burden.^33^ Nevertheless, several challenges regarding the clinical application of PSMA PET/CT remain to be addressed. PSMA PET/CT exhibits limited diagnostic accuracy for rare pathological types, such as prostatic ductal adenocarcinoma (DAC). Pepe et al. reported two cases with negative PSMA PET/CT results, which were ultimately diagnosed as a mixed pT3bN1 PCa (prostatic ductal plus acinar adenocarcinoma).^35^ Besides, determining the optimal cutoff of SUVmax in distinguishing PCa from BPD is difficult, perhaps due its semi-quantitative nature.

Therefore, radiomics is a new approach that can be used to mine the information contained in medical images and convert images into high-dimensional data via the high-throughput extraction of large numbers of imaging features.^36^ Zamboglou et al. found that radiomics of PSMA PET/CT detected PCa lesions missed by visual PET image interpretation, with a sensitivity of ≥ 0.8 in the validation cohort.^37^ However, they only enrolled PCa patients and defined non-PCa tissue as the volume of non-tumor area of the prostate gland in PCa patients. The present multicenter study enrolled patients with both PCa and BPD to comprehensively evaluate radiomics data from PSMA PET/CT, which was closer to clinical practice. Yi et al. constructed a PSMA PET-based radiomics model based on whole-gland segmentation and achieved promising diagnostic accuracy for csPCa in patients with invisible intraprostatic lesions on PSMA PET/CT.^38^ Unlike their study which focused on patients with negative PSMA-PET/CT scans, wed evaluated the performance of PET/CT radiomics for PCa detection regardless of the PET/CT report.

Our study had several limitations. First, this was a retrospective, multicenter study, which called for prospective validation in future studies to further confirm the robustness of our findings. Second, a subgroup analysis was conducted using PI-RADS scores to predict csPCa aiming to compare the performance of mpMRI and PSMA PET/CT. However, half of the enrolled patients did not undergo mpMRI in this study, which might introduce selection bias and compromise the reliability of the findings. Third, as mentioned above, the false negative rate of PSMA PET/CT in the diagnosis of DAC warrants special attention. We failed to report due to limited ductal carcinoma cases in this study and further study was needed.

## 5 Conclusion

The present multicenter study established and validated ^68^Ga-PSMA PET/CT-based radiomics models trained by the XGBoost algorithm, showing promising results and offering new insights for primary prostate cancer detection.

## CRediT authorship contribution statement

**Jiaxian Chen, Xin Jiang:** Data curation, Resources, Writing – original draft, Writing – review & editing. **Guangjie Yang, Yongxiang Tang:** Investigation, Writing – review & editing. **Lin Qi, Minfeng Chen, Shuo Hu** and **Xiaomei Gao:** Investigation, Conceptualization. **Yu Gan:** Data curation, Investigation, Writing – review & editing. **Mingxin Zhang, Shouzhen Chen** and **Yi Cai:** Conceptualization, Project administration, Funding acquisition.

## Ethics statement

This study was ethically approved by the Ethics Review Committee of Xiangya Hospital, Central South University (approval number: 202110113). Informed consent was obtained from all individual participants included in the study in three medical centers.

## Appendix Supplementary materials

Image, application 1
