# Supplementary material for: Construction and external validation of radiomics models to detect primary prostate cancer with machine learning: a multicenter study based on 68Ga-PSMA PET/CT
Source: J Natl Cancer Cent. 2026 Jan 5;6(3):297–306. doi: 10.1016/j.jncc.2025.12.002 (PMC13250527; doi:10.1016/j.jncc.2025.12.002)

Supplementary materials

Construction and external validation of radiomics models to detect primary prostate cancer with machine learning: a multicenter study based on ^{68}Ga-PSMA PET/CT

Jiaxian Chen, Xin Jiang, Guangjie Yang, Yongxiang Tang, Lin Qi, Minfeng Chen, Shuo Hu, Xiaomei Gao, Yu Gan, Mingxin Zhang, Shouzhen Chen, Yi Cai

Materials and methods

Biopsy criteria

The biopsy criteria of this study are as follows: 1.Digital rectal examination reveals a suspicious prostate nodule, regardless of prostate-specific antigen (PSA) value; 2.Suspicious lesions found by transrectal prostate ultrasound or magnetic resonance imaging (MRI), regardless of PSA value; 3.PSA >10 ng/ml; 4.PSA:4-10 ng/ml, with suspicious free/total (f/t) PSA or PSA density (PSAD) value.

mpMRI examination and image evaluation

The multi-parametric magnetic resonance imaging (mpMRI) examination consisted of T1-weighted imaging in the axial plane, T2-weighted imaging in three planes, diffusion-weighted imaging with apparent diffusion coefficient maps calculated, and dynamic contrast-enhanced imaging. The mpMRI was performed at 3 T using an external coil (Siemens Healthineers, GE). The images were evaluated in accordance with the PI-RADS v2.1 standard[1] and were interpreted by two genitourinary radiologists independently. In cases of disagreement, the final Prostate Imaging Reporting & Data System (PIRADS) score was determined through joint assessment and consensus. Prolate ellipsoid formula (length×width×height×0.52) was used to assess the prostate volume (PV) on T2-weighted images. PSAD was deffned as the ratio of PSA to PV.

Procedure of PET/CT-guided targeted biopsy

In all patients, experienced urologists performed transperineal prostate systematic biopsy (SB) with 12 cores. Further prostate-specific membrane antigen (PSMA) positron emission tomography/computed tomography (PET/CT)-guided biopsy was

performed for positive patients, and dominant lesion (defined as the highest focal uptake on PET images) was assigned 2-4 cores[2]. To identify images and targeted biopsy lesions, we used the BK Fusion Biopsy System to match labeled images with real-time prostate ultrasonography scans. All suspicious lesions were fused and targeted in real-time using transrectal ultrasound images with a biplane 6/9/12 MHz Pro Focus Transducer 8848 ultrasound system (BK Ultrasound, Peabody, MA, USA). This equipment was selected as it allows the surgeon to perform both transrectal and transperineal prostate biopsy with rigid fusion in PET/CT transrectal ultrasound fusion prostate biopsy.

Radiomics feature extraction

According to the guidelines of the Image Biomarker Standardization Initiative (IBSI), radiomic features were extracted from the volume of interest (VOI) of PSMA PET and CT images. PET images were standardized uptake value (SUV)-normalized and resampled to isotropic 1× 1× 1 mm^3 voxels with a bin width of 0.5 SUV, while CT images were resampled to isotropic 1× 1× 1 mm^3, Hounsfield unit (HU)-standardized, and discretized with a bin width of 25 HU. Two common preprocessing filters—wavelet, and Laplacian of Gaussian (LoG)—were applied. For the wavelet filter, Daubechies wavelets (db6) were employed. For the LoG filter, a scale of $\sigma = 3.0$ mm was used. All filter parameters were predefined and automatically configured within Python scripts. Overall, 2353 radiomic features (1223 features from CT images and 1130 from PET images) were extracted. The features included 450 first-order intensity statistics features, 28 shape features, and 2275 texture features (Gray Level Size Zone Matrix [GLSZM], n = 400; Gray Level Co-occurrence Matrix [GLCM], n = 600, Neighboring Gray Tone Difference Matrix [NGTDM], n = 125, Gray Level Dependence Matrix [GLDM], n =350; and Gray Level Run Length Matrix [GLRLM], n = 400).

Radiomics feature selection

For clinically significant prostate cancer (csPCa) prediction, the 12 most relevant radiomics features were selected by the minimum redundancy maximum relevance (mRMR) and least absolute shrinkage and selection operator (LASSO) algorithms. They are, respectively,

CT_lbp.3D.m2_firstorder_Range; CT_lbp.3D.m1_firstorder_Median;

PET_logarithm_glcm_ClusterTendency;
CT_lbp.3D.m1_glszm_GrayLevelNonUniformity;
PET_wavelet.LHL_firstorder_Mean;
PET_log.sigma.1.mm.3D_glcm_Correlation;
PET_wavelet.LHL_gldm_LowGrayLevelEmphasis;
PET_square_glcm_ClusterProminence;
PET_log.sigma.1.mm.3D_ngtdm_Strength;
PET_wavelet.HHH_firstorder_Skewness;
CT_lbp.3D.k_gldm_DependenceEntropy;
PET_square_gldm_DependenceVariance.

For PCa prediction, the 12 most relevant radiomics features were selected by the mRMR and LASSO algorithms. They are, respectively,
PET_logarithm_glcm_ClusterTendency
PET_log.sigma.1.mm.3D_ngtdm_Strength
PET_wavelet.LHL_firstorder_Mean
CT_lbp.3D.k_gldm_DependenceEntropy
PET_logarithm_glcm_Imc2
PET_log.sigma.1.mm.3D_firstorder_Skewness
PET_log.sigma.1.mm.3D_ngtdm_Busyness
PET_wavelet.HLH_gldm_DependenceNonUniformityNormalized
CT_log.sigma.1.mm.3D_glcm_InverseVariance
PET_log.sigma.1.mm.3D_glcm_MCC
PET_original_glcm_Imc2
PET_square_glcm_ClusterProminence.

References

1. Turkbey B, Rosenkrantz AB, Haider MA, et al. Prostate Imaging Reporting and Data System Version 2.1: 2019 Update of Prostate Imaging Reporting and Data System Version 2. *Eur Urol* 2019; **76**(3): 340-51.
2. Qiu DX, Li J, Zhang JW, et al. Dual-tracer PET/CT-targeted, mpMRI-targeted, systematic biopsy, and combined biopsy for the diagnosis of prostate cancer: a pilot study. *Eur J Nucl Med Mol Imaging* 2022; **49**(8): 2821-32.

Supplementary Table 1

Baseline clinical characteristics of different cohorts for csPCa prediction.

Characteristic	Training cohort	Internal validation cohort	External validation cohort 1	External validation cohort 2
Number of patients	175	174	192	68
Age at biopsy, mean±SD, years	66.9±7.8	66.3±8.2	67.9±8.4	69.1±8.1
PSA at biopsy, median (IQR), ng/mL	13.9 (8.2-44.8)	18.3 (9.2-38.1)	7.2 (4.5-17.8)	27.4 (12.5-95.6)
DRE, No. (%)				
Abnormal	95 (54.3)	97 (55.7)	90 (46.9)	44 (64.7)
Normal	80 (45.7)	77 (44.3)	102 (53.1)	24 (35.3)
SUVmax of dominant lesion of suspicion, median (IQR)	9.7 (5.7-18.9)	9.6 (5.1-19.5)	7.9 (4.4-19.5)	20.5 (12.3-41.3)
Pathologic results, No. (%)				
Non-csPCa (BPD and ISUP Group1)	59 (33.7)	58 (33.9)	90 (46.9)	18 (26.5)
csPCa	116 (66.3)	116 (66.1)	102 (53.1)	50 (73.5)
ISUP grade group, No. (%)				
GG1	10 (7.9)	10 (7.9)	6 (5.6)	4 (7.4)
GG2	26 (20.6)	20 (15.9)	16 (14.8)	7 (13.0)
GG3	17 (13.5)	16 (12.7)	19 (17.6)	4 (7.4)
GG4	23 (18.3)	29 (23.0)	32 (29.6)	19 (35.2)
GG5	50 (39.7)	51 (40.5)	35 (32.4)	20 (37.0)
Primary Gleason score, No. (%)				
3	43 (34.1)	32 (25.4)	22 (20.4)	11 (20.4)
4	51 (40.5)	60 (47.6)	57 ((52.8)	34 (62.9)
5	32 (25.4)	34 (27.0)	29 (26.8)	9 (16.7)

Secondary Gleason score, No. (%)				
3	29 (23.0)	31 (24.6)	25 (23.1)	8 (14.8)
4	55 (43.7)	58 (46.0)	72 (66.7)	31 (57.4)
5	42 (33.3)	37 (29.4)	11 (10.2)	15 (27.8)

Abbreviations: BPD, benign prostate diseases; csPCa, clinically significant prostate cancer; DRE, digital rectal examination; GG, grade group; IQR, interquartile range; ISUP, International Society of Urological Pathology; PSA, prostate-specific antigen; SD, standard deviation; SUVmax, maximum standardized uptake value.

Supplementary Table 2

Baseline clinical characteristics of different cohorts for PCa prediction.

Characteristic	Training cohort	Internal validation cohort	External validation cohort 1	External validation cohort 2
Number of patients	175	174	192	68
Age at biopsy, mean±SD, years	66.9±7.9	66.3±8.1	67.9±8.4	69.1±8.1
PSA at biopsy, median (IQR), ng/mL	16.4 (9.0-39.1)	15.6 (8.1-40.5)	7.2 (4.5-17.8)	27.4 (12.5-95.6)
DRE, No. (%)				
Abnormal	92 (52.6)	100 (57.5)	90 (46.9)	44 (64.7)
Normal	83 (47.4)	74 (42.5)	102 (53.1)	24 (35.3)
SUVmax of dominant lesion of suspicion, median (IQR)	9.7 (5.2-18.9)	9.5 (5.5-19.1)	7.9 (4.4-19.5)	20.5 (12.3-41.3)
Pathologic results, No. (%)				
BPD	49 (28)	48 (27.6)	84 (43.7)	14 (20.6)
PCa	126 (72)	126 (72.4)	108 (56.3)	54 (79.4)
ISUP grade group, No. (%)				
GG1	9 (7.1)	11 (8.7)	6 (5.6)	4 (7.4)
GG2	25 (19.8)	21 (16.7)	16 (14.8)	7 (13.0)
GG3	18 (14.3)	15 (11.9)	19 (17.6)	4 (7.4)
GG4	23 (18.3)	29 (23.0)	32 (29.6)	19 (35.2)
GG5	51 (40.5)	50 (39.7)	35 (32.4)	20 (37.0)
Primary Gleason score, No. (%)				
3	36 (28.6)	39 (31.0)	22 (20.4)	11 (20.4)
4	55 (43.7)	56 (44.4)	57 ((52.8)	34 (62.9)
5	35 (27.7)	31 (24.6)	29 (26.8)	9 (16.7)

Secondary Gleason score, No. (%)				
3	31 (24.6)	29 (23.0)	25 (23.1)	8 (14.8)
4	61 (48.4)	52 (41.3)	72 (66.7)	31 (57.4)
5	34 (27.0)	45 (35.7	11 (10.2)	15 (27.8)

Abbreviations: BPD, benign prostate diseases; DRE, digital rectal examination; GG, grade group; IQR, interquartile range; ISUP, International Society of Urological Pathology; PCa, prostate cancer; PSA, prostate-specific antigen; SD, standard deviation; SUVmax, maximum standardized uptake value.

Supplementary Table 3

Baseline clinical characteristics of patients who completed mpMRI examination.

**Characteristic**	**Center1**	**Center2**
Number of patients	189	192
Age at biopsy, mean±SD, years	66.9±7.5	67.9±8.4
PSA at biopsy, median (IQR), ng/mL	13.1 (7.6-27.6)	7.2 (4.5-17.8)
Volume, median (IQR), mL	38.4 (26.7-59.9)	43.5 (28.3-63.0)
PSAD, median (IQR), ng/ml^2	0.32 (0.2-0.8)	0.20 (0.1-0.4)
DRE, No. (%)		
Abnormal	114 (60.3)	90 (46.9)
Normal	75 (39.7)	102 (53.1)
mpMRI results, No. (%)		
PI-RADS 1-2	9 (4.8)	54 (28.1)
PI-RADS 3	73 (38.6)	44 (22.9)
PI-RADS 4	23 (12.2)	52 (27.1)
PI-RADS 5	84 (44.4)	42 (21.9)
Pathologic results, No. (%)		
BPD	68 (36.0)	84 (43.7)
PCa	121 (64.0)	108 (56.3)
ISUP grade group, No. (%)		
GG 1	13 (10.7)	6 (5.6)
GG 2	27 (22.3)	16 (14.8)
GG 3	16 (13.2)	19 (17.6)
GG 4	23 (19.0)	32 (29.6)
GG 5	42 (34.7)	35 (32.4)

Abbreviations: BPD, benign prostate diseases; DRE, digital rectal examination; GG, grade group; IQR, interquartile range; ISUP, International Society of Urological Pathology; mpMRI, multi-parameter magnetic resonance imaging; PCa, prostate cancer; PI-RADS, Prostate Imaging Reporting and Data System; PSA, prostate-specific antigen; PSAD, prostate specific antigen density; SD, standard deviation; SUVmax, maximum standardized uptake value.

Supplementary Fig. 1. Flowchart of the patient selection process. ^{68}Ga-PSMA, ^{68}Ga-prostate-specific membrane antigen; BPD, benign prostatic hyperplasia; PCa, prostate cancer; PET/CT, positron emission tomography/computed tomography.

Supplementary Fig. 2. The SHAP analysis of the radiomics model developed by the XGBoost algorithm for clinical significant prostate cancer prediction. (A) The SHAP bar chart shows the weight of the 12 selected radiomics features in the model. (B) The SHAP bees-warm plot shows the effects of each selected feature on the prediction probability. SHAP, SHapley Additive exPlanations.

Supplementary Fig. 3. Confusion Matrix analysis of the combined model across different cohorts for clinical significant prostate cancer prediction. (A) Training cohort. (B) Internal validation cohort. (C) External validation cohort 1. (D) External validation cohort 2.

Supplementary Fig. 4. The SHAP analysis of the radiomics model developed by the XGBoost algorithm for prostate cancer prediction. (A) The SHAP bar chart shows the weight of the 12 selected radiomics features in the model. (B) The SHAP bees-warm plot shows the effects of each selected feature on the prediction probability. SHAP, SHapley Additive exPlanations.

Supplementary Fig. 5. Confusion Matrix analysis of the combined model across different cohorts for prostate cancer prediction. (A) Training cohort. (B) Internal validation cohort. (C) External validation cohort 1. (D) External validation cohort 2.

Supplementary FIg. 1

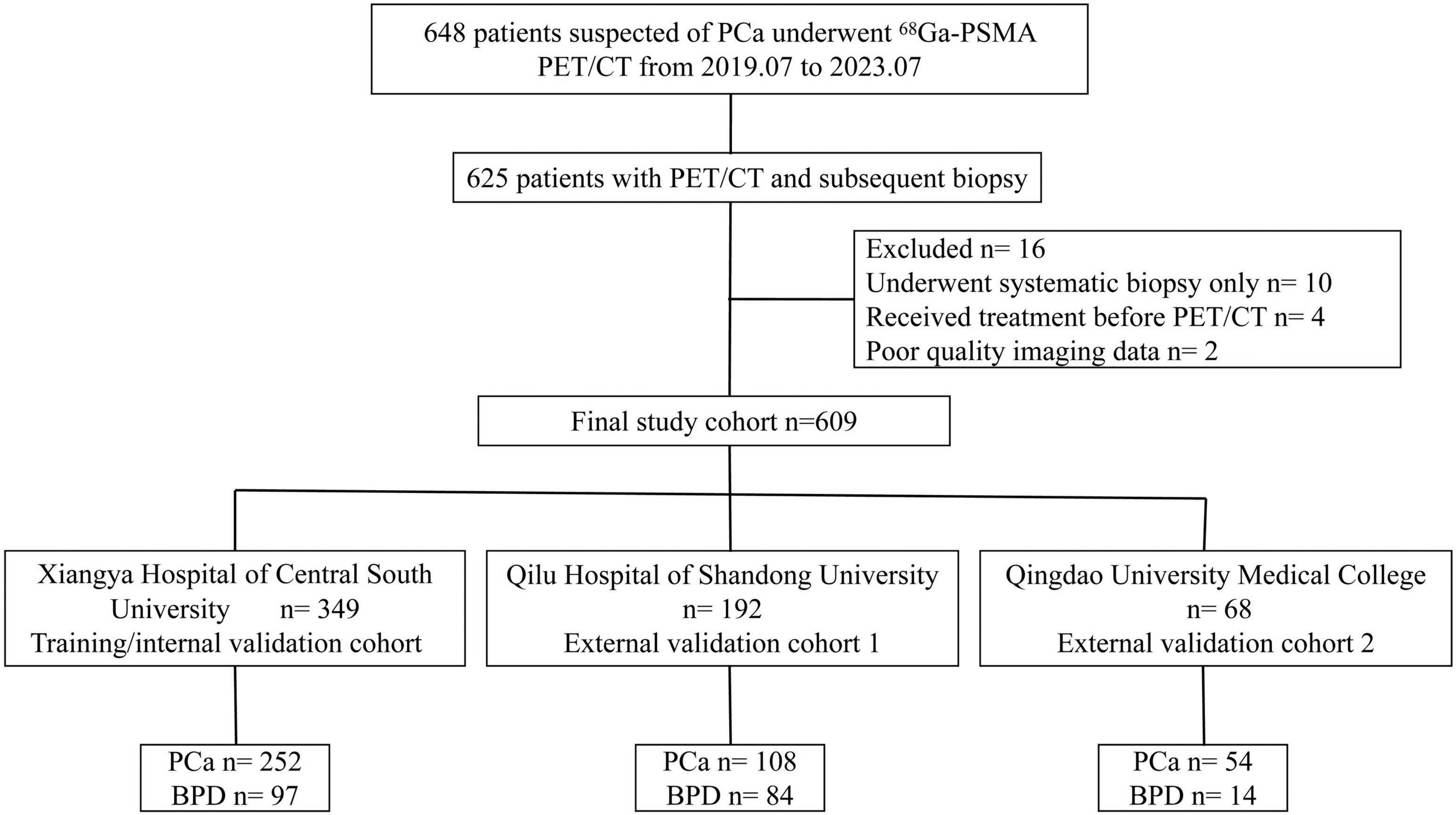

Supplementary FIg. 2

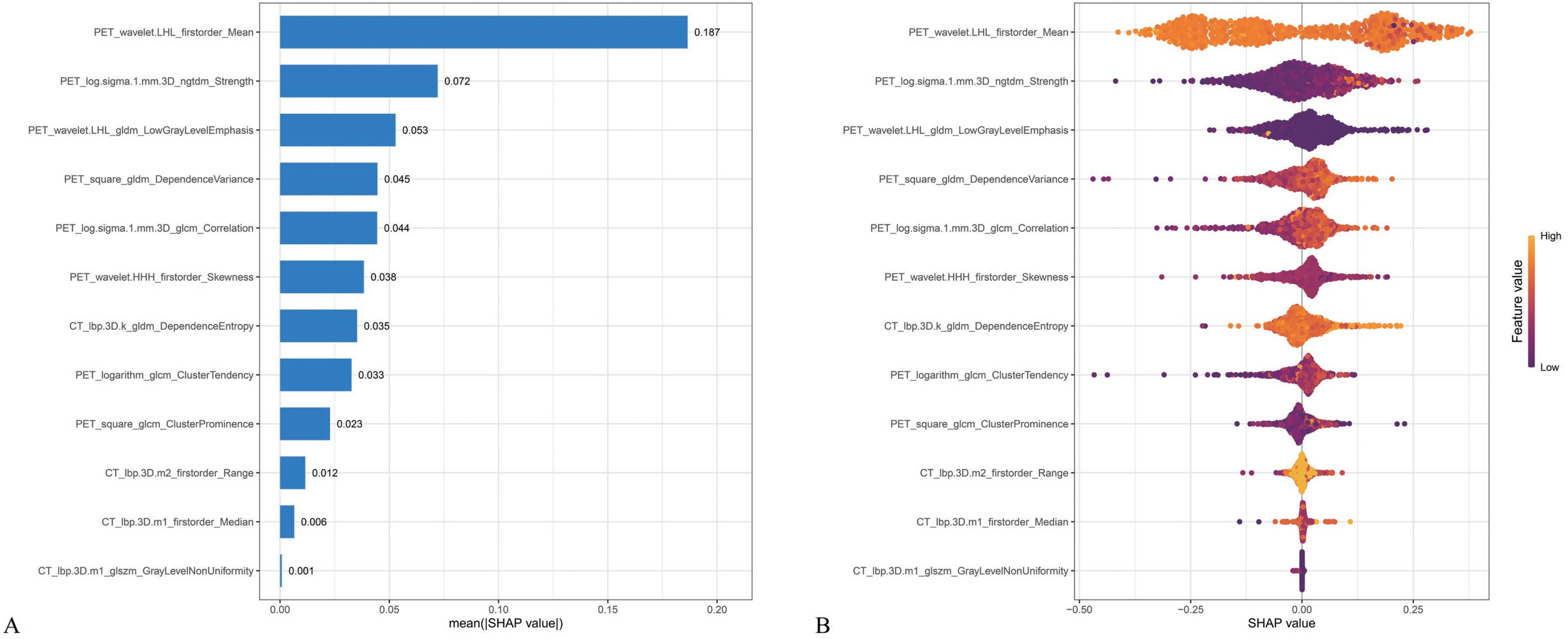

Supplementary FIg. 3

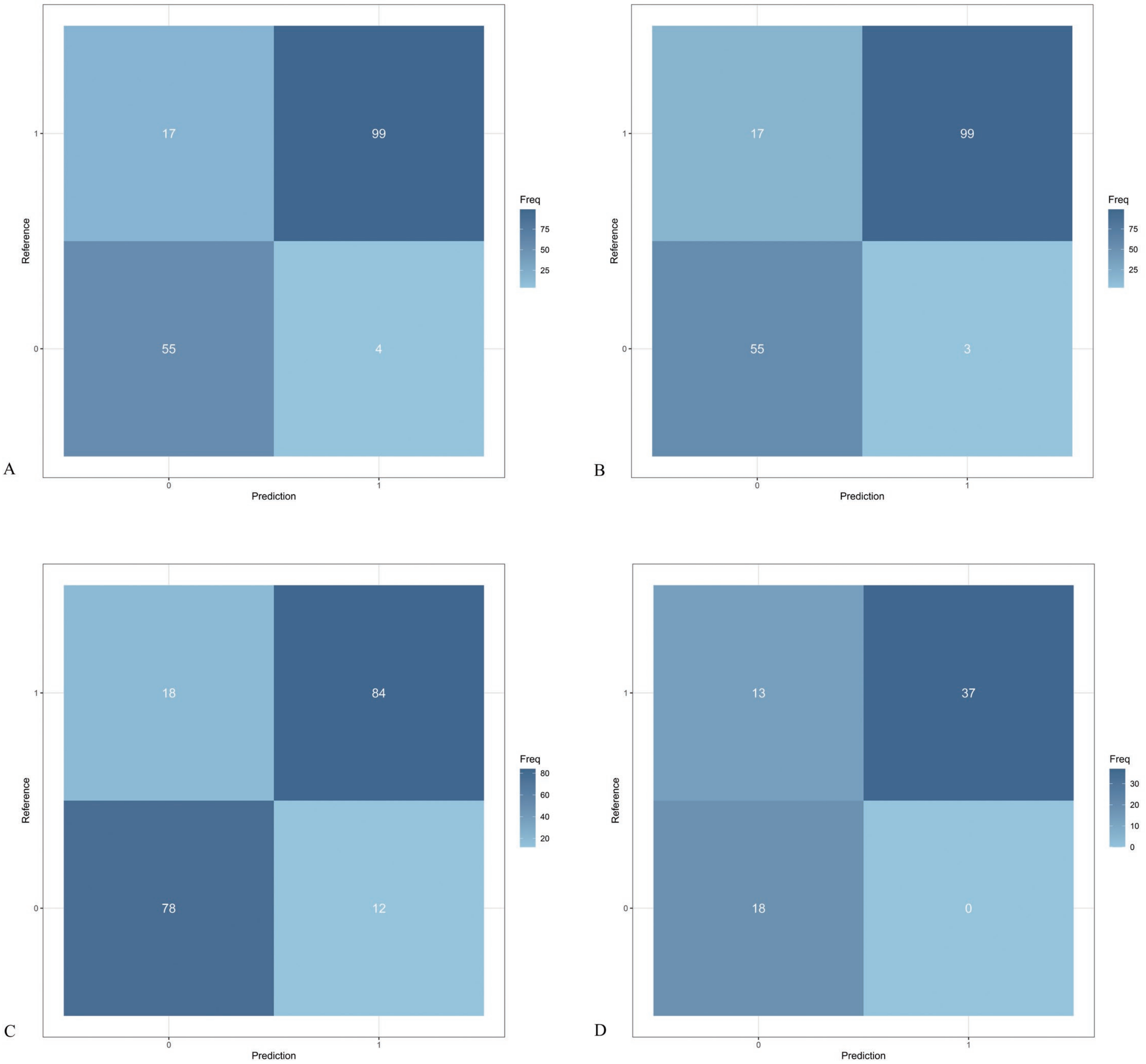

Supplementary FIg. 4

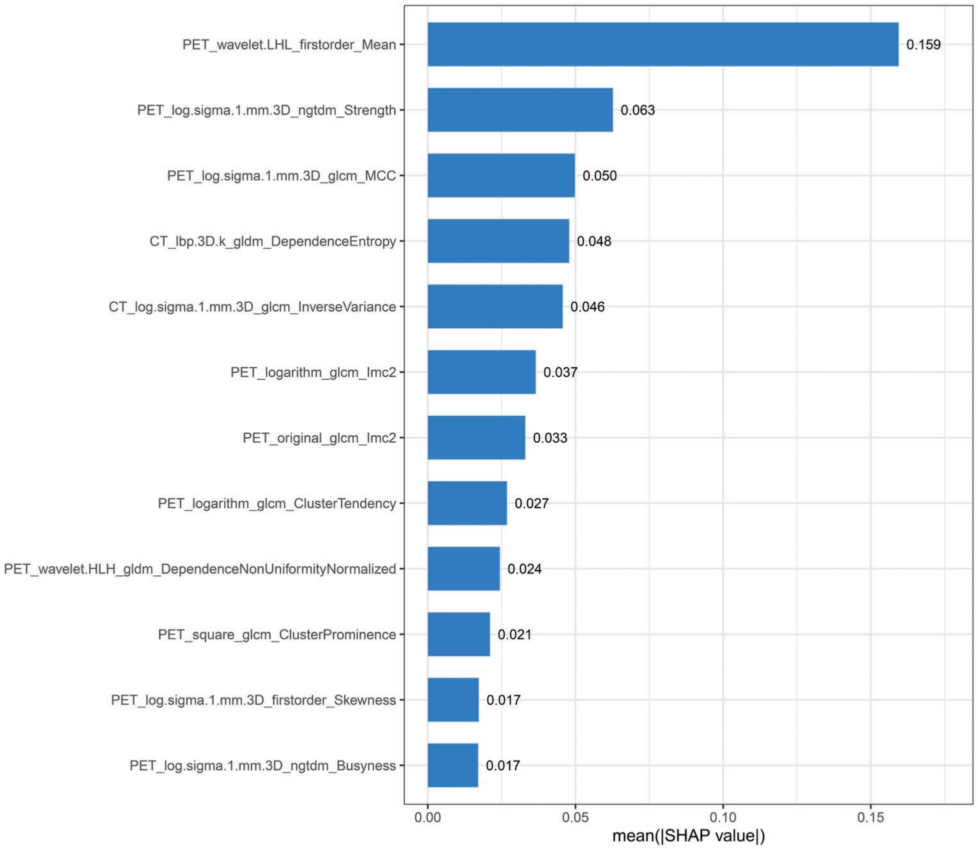

A

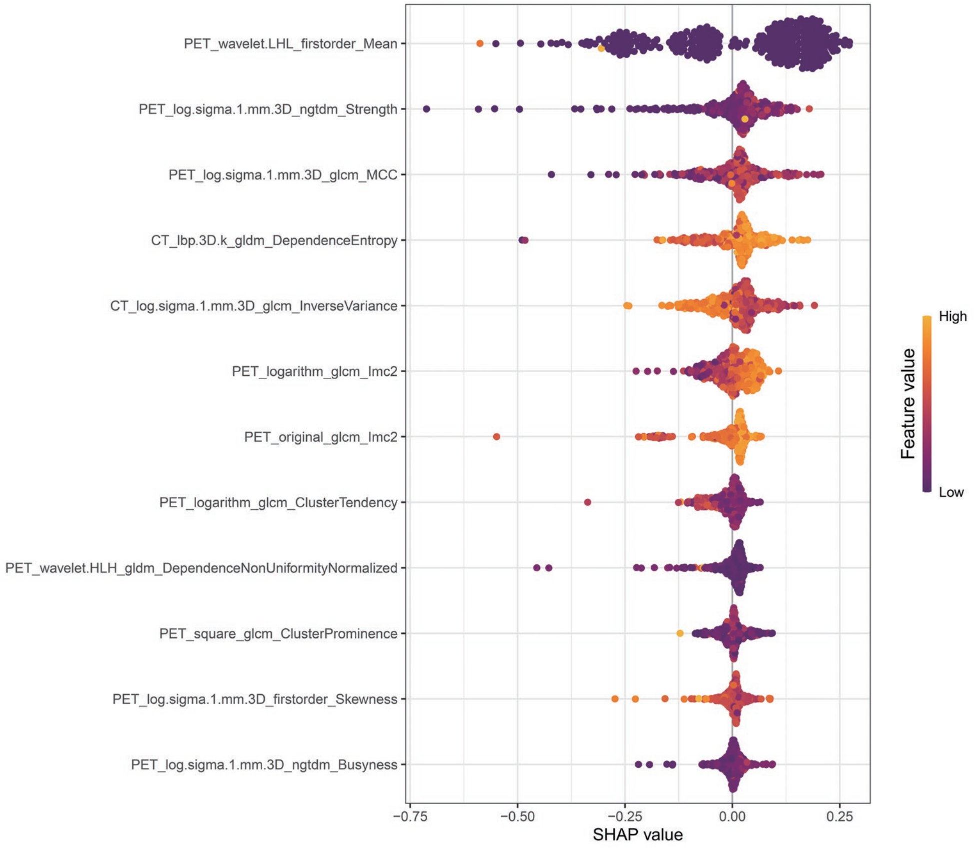

B

Supplementary FIg. 5

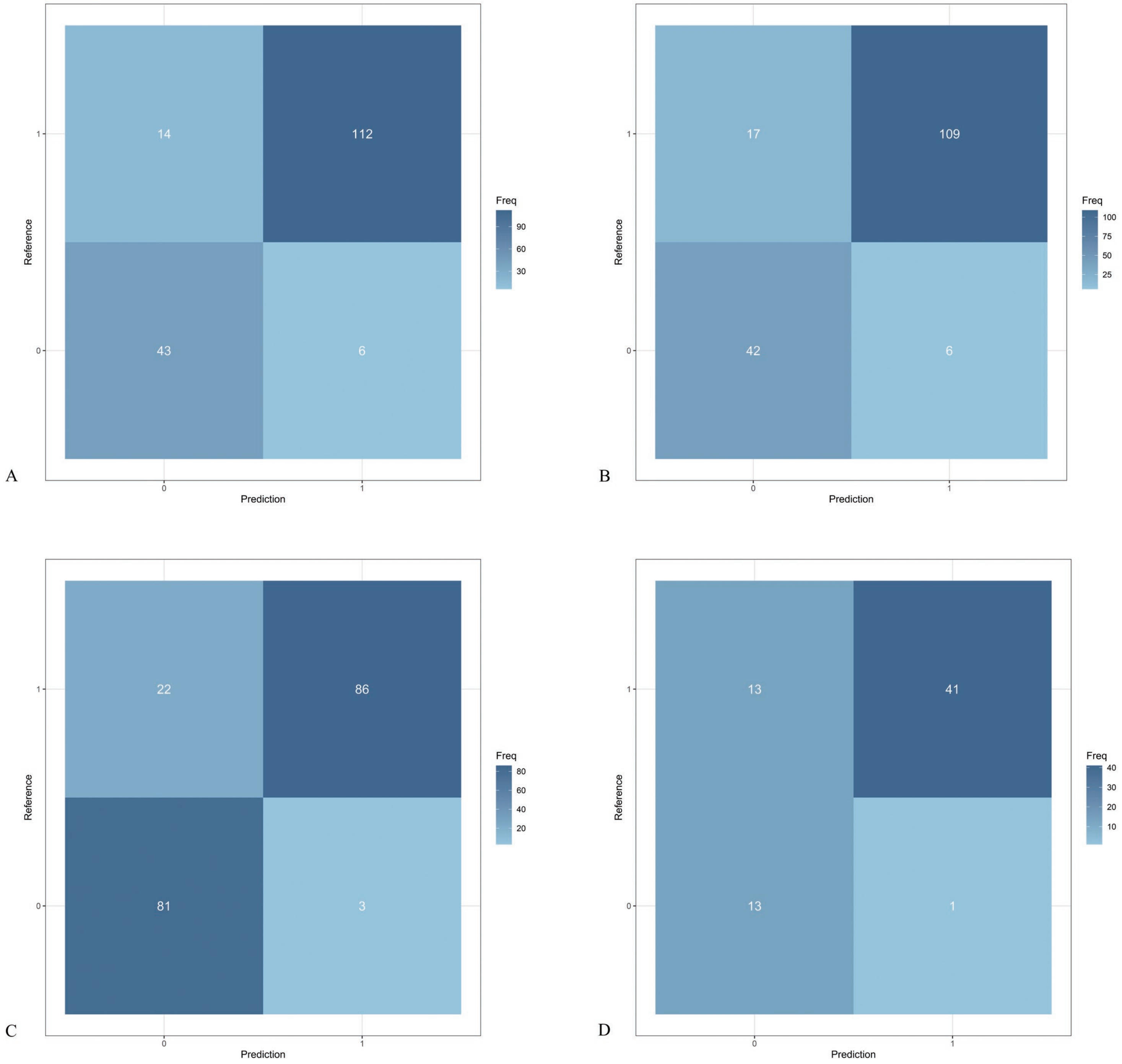

Supplement: Supplementary file 1 [file mmc1.pdf]
